# Complete Genome Sequence of Serratia marcescens Podophage Parlo

**DOI:** 10.1128/MRA.00569-19

**Published:** 2019-07-03

**Authors:** Ryan Bockoven, Jamie Gutierrez, Heather Newkirk, Mei Liu, Jesse Cahill, Jolene Ramsey

**Affiliations:** a Center for Phage Technology, Texas A&M University, College Station, Texas, USA; DOE Joint Genome Institute

## Abstract

Serratia marcescens is an opportunistic human pathogen with multiple resistance mechanisms that infects hospitalized patients. Here, we report the full genome sequence of S. marcescens podophage Parlo. Parlo is most similar to Erwinia phage PEp14 and encodes a 3,764-residue protein assumed to be a homolog of DarB, an antirestriction protein.

## ANNOUNCEMENT

Serratia marcescens is a Gram-negative opportunistic human pathogen (1). It is found within many environments and is well known for infecting hospitalized patients. S. marcescens has resistance to certain penicillins, cephalosporins, tetracyclines, and other antibiotics (2). Thus, we investigated bacteriophages of S. marcescens as potential treatment alternatives and report here the genome sequence of podophage Parlo.

Parlo grows on Serratia marcescens D1 (Ward’s Science, catalog no. 8887172) and was isolated from pooled swine, fecal, and soil samples collected at private farms primarily around Bryan and College Station, Texas. These solid samples were mixed with LB medium, and then supernatants were sterilized by filter (0.22 μm) or chloroform. Hosts and phages were cultured at 30°C in LB broth and agar (BD) as previously described (3). Phage morphology was determined using a JEOL 1200EX transmission electron microscope in the Texas A&M Microscopy and Imaging Center to observe samples negatively stained with 2% (wt/vol) uranyl acetate (4). Phage genomic DNA was purified with the Promega Wizard DNA cleanup kit according to the modification by Summer (5), and an Illumina TruSeq Nano low-throughput (LT) kit was used to generate the library for sequencing using v2 500-cycle chemistry on an Illumina MiSeq instrument with 250-bp paired-end reads. Quality checks and trimming were performed on the 373,956 total reads using FastQC (http://www.bioinformatics.babraham.ac.uk/projects/fastqc/) and the FastX Toolkit 0.0.14 (http://hannonlab.cshl.edu/fastx_toolkit/). A single circularized contig with 395-fold coverage was assembled with SPAdes 3.5.0 using default parameters (6). Sanger sequencing of PCR products from the ends of the genome (forward, 5′-CATAAACCAGCAGCTGCAAAC-3′; reverse, 5′-TCCAGTTGCATGATCGGTTAG-3′) verified that the assembly was complete. Gene locations were determined using GLIMMER 3.0 and MetaGeneAnnotator 1.0 or ARAGORN 2.36 for tRNA genes (7–9). Gene functional prediction was performed using InterProScan 5.22, LipoP, TMHMM, and BLASTp 2.2.31 results against those of the UniProtKB Swiss-Prot/TrEMBL, and NCBI nonredundant (nr) databases (10–14). TransTerm was used to detect rho-independent termination sites (http://transterm.cbcb.umd.edu/). All tools were hosted on a Galaxy server (https://cpt.tamu.edu/galaxy-pub/) by the Center for Phage Technology at Texas A&M University, and annotation was performed using Web Apollo (15).

Podophage Parlo has a 61,626-bp genome with 87 predicted protein-encoding genes at an average length of 717 bp, 30 of which have a predicted function. Phage Parlo has a GC content of 58.0% and a 95.8% coding density. No tRNAs were identified. Parlo is predicted by PhageTerm (16) to use a *pac*-type headful packaging mechanism.

Parlo encodes a 3,764-residue-long hypothetical protein (GenBank accession no. QBQ72230). The corresponding gene matches several InterProScan domains, including helicase domains (e.g., InterProScan domain IPR027417), methyltransferase domains (IPR029063), an N-terminal transglycosylase domain (IPR008258), and acyltransferase domains (e.g., IPR000182). These domains were previously observed in DarB homologs of Bcep22-like phages, which suggests that this protein is a homolog of phage P1 DarB (GenBank accession no. YP_006479), an antirestriction protein that protects phage DNA upon infection (17). Using progressiveMauve 2.4.0 (18), we compared Parlo to other phages and found it to be most similar to *Erwinia* phage PEp14 (GenBank accession no. JN585957), with only 33 similar proteins and 20.2% nucleotide similarity. Parlo also shares similarities (19 to 21 proteins and 7.2 to 7.4% nucleotide identities) with three *Burkholderia* sp. phages, BcepIL02 (FJ937737), Bcepmigl (JX104231), and DC1 (JN662425). Like Parlo, BcepIL02 possesses a DarB homolog. Parlo has a class III holin and embedded spanin genes for lysis.

### Data availability.

The genome sequence and associated data for phage Parlo were deposited in GenBank under the accession no. MK618715, BioProject no. PRJNA222858, SRA no. SRR8869229, and BioSample no. SAMN11360383.
